# First-Trimester Serum Omentin Levels as a Predictor of Gestational Diabetes Mellitus

**DOI:** 10.7759/cureus.110173

**Published:** 2026-06-03

**Authors:** Abhigya Malik, Sheeba Marwah, Megha Arora, Nidhi Verma, Drashya Khatri, Archana B. S., Amandeep Kaur, Muskan Bhalla, Bindu Bajaj

**Affiliations:** 1 Department of Obstetrics and Gynaecology, Vardhman Mahavir Medical College and Safdarjung Hospital, New Delhi, IND; 2 Department of Biochemistry, Vardhman Mahavir Medical College and Safdarjung Hospital, New Delhi, IND

**Keywords:** adipokines, first trimester, gestational diabetes mellitus, ogtt, omentin, predictive biomarkers

## Abstract

Background: Gestational diabetes mellitus (GDM) is a frequent metabolic complication of pregnancy associated with significant maternal and neonatal morbidity. Its prevalence is notably higher among South Asian women, likely due to increased baseline insulin resistance and genetic predisposition. Conventional screening using the oral glucose tolerance test (OGTT), typically performed at 24-28 weeks of gestation, often identifies GDM after metabolic alterations are already established, thereby limiting opportunities for early intervention. This has led to growing interest in identifying reliable first-trimester biomarkers for early risk prediction. Adipokines, which play a crucial role in metabolic regulation and inflammation, have emerged as promising candidates. Omentin, an insulin-sensitizing adipokine predominantly secreted by visceral adipose tissue, has been implicated in glucose homeostasis. This study aimed to evaluate first-trimester serum omentin levels as a predictor of GDM and to assess its association with fetomaternal outcomes.

Methods: A prospective cohort study was conducted over a period of 18 months from July 2024 to December 2025 at Vardhman Mahavir Medical College (VMMC) and Safdurjung Hospital. Pregnant women aged 18-35 years with a singleton pregnancy and a normal two-hour OGTT at their first antenatal visit were enrolled. First-trimester venous blood samples were collected under standardized conditions, and serum omentin levels were measured using enzyme-linked immunosorbent assay (ELISA). Participants underwent repeat 75-g OGTT at 24-28 weeks and again at 32-34 weeks of gestation. All participants were followed until delivery to monitor the development of GDM and to document relevant maternal and neonatal outcomes.

Results: Lower first-trimester serum omentin levels were significantly associated with subsequent development of GDM and demonstrated moderate predictive utility on receiver operating characteristic curve (ROC) analysis, with an area under the receiver operating characteristic curve (AUROC) of 0.683. At the optimal cutoff value, specificity was 83%, and the negative predictive value was 90.8%, indicating a strong capacity to identify women at low risk for developing GDM. A trend toward increased adverse fetomaternal outcomes was observed among women with lower omentin levels.

Conclusions: First-trimester serum omentin shows promise as an early predictive biomarker for GDM. Its incorporation into routine antenatal screening protocols may improve early risk stratification and facilitate timely preventive interventions. These findings support the potential role of adipokine-based screening strategies in enhancing current approaches to GDM detection and improving overall pregnancy outcomes.

## Introduction

Gestational diabetes mellitus (GDM) is described as glucose intolerance of varying severity that is first established during pregnancy. According to the American Diabetes Association, it refers to diabetes diagnosed in the second or third trimester in women without prior overt diabetes [1]. It is the most common metabolic disorder of pregnancy and remains a major challenge in perinatal medicine [2].

The burden of GDM is considerable and continues to rise worldwide. Reported prevalence ranges from 1% to 30% depending on population characteristics, diagnostic criteria, and screening methods. On average, global prevalence is estimated at around 14%, with particularly high rates in Africa, Asia, and South Asia, including India [1-3]. This variability reflects genetic predisposition, lifestyle factors, and differences in screening practices, but the overall trend points to a growing public health concern. GDM is associated not only with adverse pregnancy outcomes but also with long-term health risks for both mother and child, making it a significant contributor to maternal and neonatal morbidity.

Screening for GDM is essential because hyperglycemia during pregnancy is linked to a wide range of complications. Mothers are at increased risk of pre-eclampsia, hypertensive disorders, operative delivery, and progression to type 2 diabetes mellitus later in life [1]. Infants born to mothers with GDM face risks such as macrosomia, hypoglycemia, hypocalcemia, hyperbilirubinemia, and even long-term metabolic disturbances [3]. Early identification of women at risk allows timely intervention, which can reduce both immediate and future health consequences. This highlights the importance of predictive biomarkers that can detect GDM susceptibility early in pregnancy.

Understanding the pathogenesis of GDM is the key to preventing its development during pregnancy, which is complex and multifactorial. Pregnancy is a state of progressive insulin resistance, driven largely by placental hormones and metabolic adaptations. In healthy women, pancreatic β-cells compensate by increasing insulin secretion to maintain glucose homeostasis. In GDM, however, this compensatory mechanism is inadequate due to β-cell dysfunction, resulting in maternal hyperglycemia [4]. Recent evidence suggests that adipokines - bioactive molecules secreted by adipose tissue and the placenta - play a significant role in modulating insulin sensitivity and may contribute to the development of GDM. Dysregulation of adipokine secretion has been linked to insulin resistance, inflammation, and altered maternal-fetal metabolic adaptation.

Given adipokines' role in insulin sensitivity, there is growing interest in their potential as early biomarkers for GDM. While molecules such as adiponectin and leptin have been extensively studied, data on newer adipokines, including apelin, chemerin, and omentin, are limited and inconsistent. Identifying reliable markers in early pregnancy could transform screening practices and improve outcomes, making omentin a promising candidate for investigation [5].

Omentin, also known as intelectin-1, is a glycoprotein of 313 amino acids primarily secreted by visceral adipose tissue, but it is also expressed in the placenta, ovaries, and heart. Two isoforms have been described - omentin-1 and omentin-2 - with omentin-1 being the predominant circulating form [5]. Omentin has insulin-sensitising properties, is inversely correlated with obesity, and is positively associated with adiponectin levels [6,7]. Beyond glucose metabolism, it has been implicated in cardiovascular health, lipid regulation, and inflammatory pathways.

In pregnancy, omentin may play a dual role: contributing to the pathogenesis of GDM by influencing insulin resistance and serving as a potential exploratory biomarker for early prediction of the condition. However, existing studies report variable associations between serum omentin levels and gestational hyperglycemia, leaving its precise role unclear. This study, therefore, aims to evaluate serum omentin-1 levels in pregnant women with and without GDM and to explore their potential utility as an early predictive biomarker.

## Materials and methods

This prospective cohort study was conducted in the Department of Obstetrics and Gynaecology between July 2024 and December 2025. The study aimed to evaluate whether serum omentin levels measured in early pregnancy could predict the subsequent development of GDM. Approval was obtained from the Institutional Ethics Committee prior to initiation, and all participants provided written informed consent before enrollment.

Study population and eligibility

Pregnant women aged 18-35 years presenting in the first trimester and attending the antenatal clinic were considered for inclusion. Only those with a normal glucose status at booking, confirmed by a two-hour oral glucose tolerance test (OGTT), were included in the study.

Women were excluded if they had a body mass index (BMI) greater than 30 kg/m^2^, a personal or family history of diabetes mellitus, a history of macrosomia or instrumental delivery in a previous pregnancy, or pre-existing medical conditions such as chronic hypertension, liver disease, or renal disorders. These criteria were applied to minimize confounding factors that could independently influence glucose metabolism.

Study flow and participant enrollment

**Figure 1:**
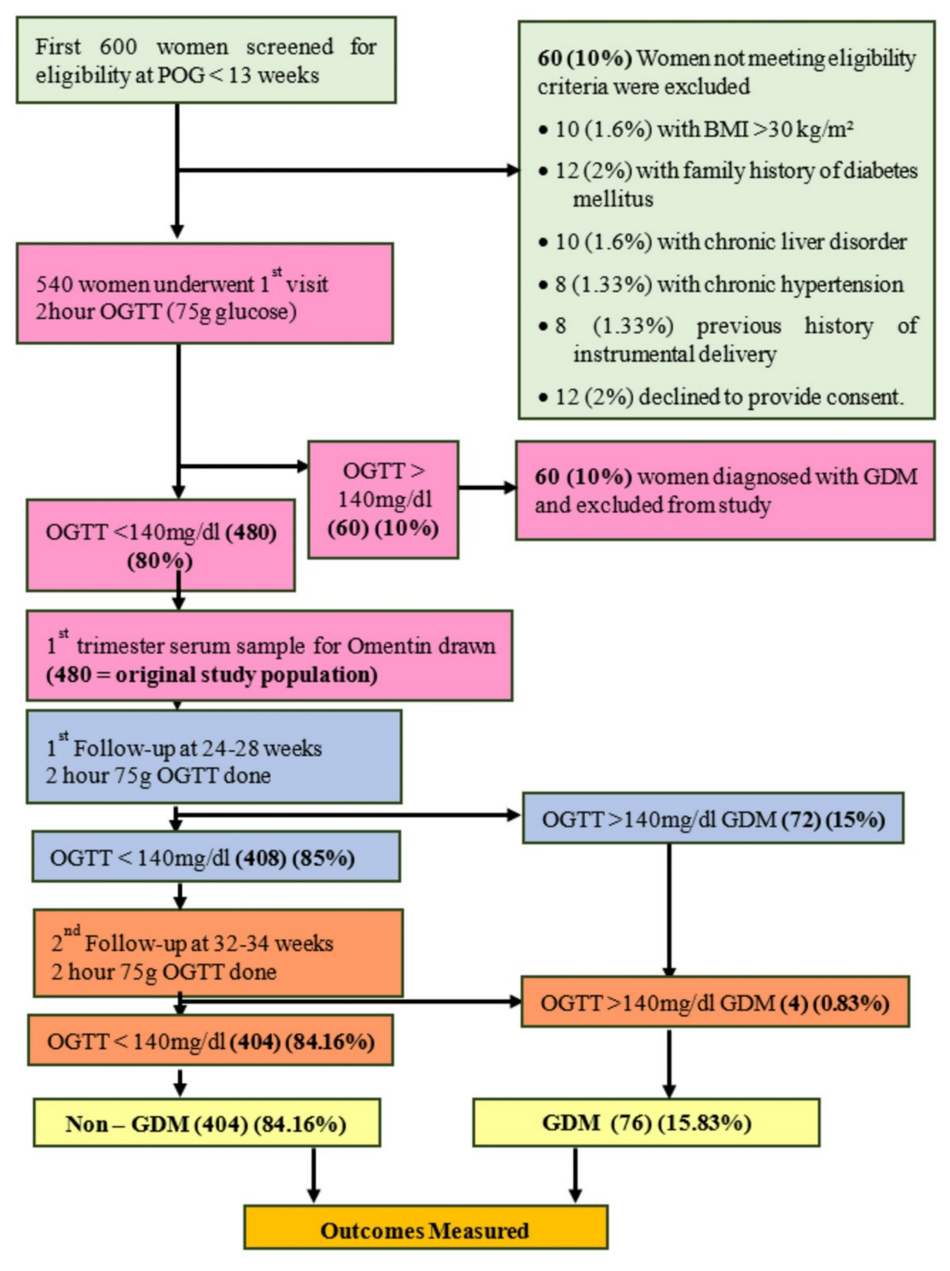
Study flow diagram GDM: gestational diabetes mellitus; OGTT: oral glucose tolerance test; POG: period of gestation; BMI: body mass index

Over the 18-month study period, 600 pregnant women in the first trimester were screened. Of these, 60 (10%) were excluded based on predefined criteria, including obesity (n=10) (1.6%), family history of diabetes (n=12) (2%), chronic liver disease (n=10) (1.6%), chronic hypertension (n=8) (1.33%), prior instrumental delivery (n=8) (1.33%), and refusal to participate (n=12) (2%). A further 60 (10%) women were excluded as they were diagnosed with GDM at booking, defined by a two-hour plasma glucose level ≥140 mg/dL following a 75-g OGTT. After exclusions, 480 (80%) women were enrolled and followed longitudinally (Figure 1).

Clinical and laboratory assessment

Baseline demographic and clinical details were recorded at enrolment using a structured data collection form (see Appendices). Under aseptic conditions, 5 mL of venous blood was drawn from each participant into plain vacutainers using sterile technique. Samples were transported within one hour under maintained cold-chain conditions using insulated containers with ice packs. Following centrifugation, serum was separated and stored at -80°C until further processing.

Serum omentin concentrations were measured using commercially available sandwich enzyme-linked immunosorbent assay (ELISA) kits (containing a human omentin antibody, a microtitre plate, biotinylated omentin antibody, streptavidin-horseradish peroxidase (HRP)) according to the manufacturer's protocols. To maintain analytical consistency and reduce inter-assay variability, samples were processed in batches of 80.

Follow-up and outcome measures

All participants received routine antenatal care and follow-up in accordance with institutional protocols. OGTT for all patients was done at the booking visit, during which 60 were diagnosed and excluded from the study. A repeat 75-g, two-hour OGTT was performed between 24 and 28 weeks of gestation, during which 72 (15%) participants were diagnosed with GDM based on the Diabetes in Pregnancy Study Group India (DIPSI) criteria (plasma glucose ≥140 mg/dL). A subsequent OGTT at 32-34 weeks identified an additional four (0.83%) cases. Participants were followed until delivery and discharge, and maternal and neonatal outcomes were documented in a predesigned patient proforma.

For analytical purposes, participants were categorized into two groups: those who developed GDM (n=76) (15.83%) and those who remained normoglycemic throughout pregnancy (n=404) (84.16%).

Sample size consideration

The sample size for this study was determined based on feasibility within the study duration and patient load of the institution. A total of 480 participants were included after applying eligibility criteria and exclusions, which was considered adequate to detect clinically meaningful differences in serum omentin levels between groups and to perform subgroup analyses with sufficient statistical power.

Statistical analysis

Data were entered and analyzed using IBM SPSS Statistics for Windows, Version 31 (Released 2025; IBM Corp., Armonk, New York, United States). Continuous variables were summarized as mean±standard deviation or median with interquartile range, depending on the distribution. Categorical variables were expressed as frequencies and percentages. Serum omentin levels were entered into the logistic regression model as a continuous variable.

Comparisons between groups were performed using independent or paired t-tests for normally distributed variables, while non-parametric data were analyzed using the Wilcoxon test. Associations between categorical variables were assessed using the chi-square test or Fisher’s exact test as appropriate. Correlation analysis was carried out using Pearson’s or Spearman’s coefficients.

Receiver operating characteristic (ROC) curve analysis was used to evaluate the predictive performance of serum omentin levels and to determine optimal cutoff values. A p-value of <0.05 was considered statistically significant.

## Results

GDM was diagnosed in 15.8% (n=76) of the study population. The majority of cases (72 out of 76; 94.7%) were detected during routine screening at 24-28 weeks of gestation, while a smaller proportion (four women; 5.3%) were identified later at 32-34 weeks. Among those diagnosed at 24-28 weeks, the mean gestational age at diagnosis was 25.32 ± 1.09 weeks. The mean OGTT value in the GDM group was significantly elevated (156.58±24.40 mg/dL; W=7327.5, p<0.001). Similarly, cases of late-onset GDM also showed significantly higher OGTT values (W=4742.5, p=0.021), with diagnosis occurring at a mean gestational age of 33.12±0.53 weeks.

In terms of demographic characteristics, the largest proportion of participants (n=248) (51.7%) belonged to the 18-25-year age group. The mean age was comparable between women with and without GDM, at 25.71±3.78 years and 25.42±3.13 years, respectively. Although women who developed GDM were marginally older, this difference did not reach statistical significance (W=4145.0, p=0.433).

The mean BMI of the study population was 23.13±2.87 kg/m^2^, with values ranging from 16.8 to 29.8 kg/m^2^. Approximately half of the participants (n=242) (50.4%) fell within the normal BMI category (18.5-22.9 kg/m^2^), making it the most prevalent group. A relatively greater proportion of women in the GDM group were classified as overweight (25.0-29.9 kg/m^2^), whereas normal BMI was more common among those without GDM. However, this difference was not statistically significant (χ^2^=5.889, p=0.106) (Table 1).

**Table 1:** Maternal and baseline characteristic of the study population GDM: gestational diabetes mellitus; SD: standard deviation

Characteristic	Total (n=480)	GDM (n=76)	Non-GDM (n=404)	p-value
Age (years)				
Mean±SD	25.47±3.24	25.71±3.78	25.42±3.13	0.433
Gravidity, n (%)				
Primigravida	188 (39.2)	26 (34.2)	162 (40.1)	0.495
Multigravida	292 (60.8)	50 (65.8)	242 (59.9)	
Body mass index (kg/m^2^)				
Mean ± SD	23.13±2.87	23.56±2.64	23.05±2.90	0.106
<18.5, n (%)	10 (2.1)	2 (2.6)	8 (2.0)	
18.5-22.9, n (%)	242 (50.4)	34 (44.7)	208 (51.5)	
23.0-24.9, n (%)	108 (22.5)	10 (13.2)	98 (24.3)	
25.0-29.9, n (%)	120 (25.0)	30 (39.5)	90 (22.3)	
Timing of GDM diagnosis, n (%)				
Diagnosed at 24-28 weeks	-	72 (94.7)	-	<0.001
Diagnosed at 32-34 weeks	-	4 (5.3)	-	0.021

Omentin levels did not follow a normal distribution across the GDM and non-GDM groups; therefore, comparisons were performed using the Wilcoxon-Mann-Whitney U test. Women who subsequently developed GDM had lower first-trimester serum omentin levels than those who remained normoglycemic. The mean (±SD) omentin level was 104.53±94.42 ng/mL in the GDM group and 139.29±89.51 ng/mL in the non-GDM group. Corresponding median (IQR) values were 64.5 (46.25-138) and 121.5 (87-167.25), respectively. The observed range of values extended from 28 to 492 in the GDM group and from 6.2 to 792 in the non-GDM group.

This difference between groups was statistically significant (W=2431.5, p<0.001), with consistently higher omentin levels observed among women who did not develop GDM. The distribution pattern also reflected a downward shift in omentin values in the GDM group. The strength of association, assessed using point-biserial correlation, was modest (r=0.14), indicating a small effect size (Table 2).

**Table 2:** Association between omentin and GDM GDM: gestational diabetes mellitus

Omentin levels	GDM		Wilcoxon-Mann-Whitney U test	
	Yes	No	W	p-value
Mean (SD)	104.53 (94.42)	139.29 (89.51)	2431.500	<0.001
Median (IQR)	64.5 (46.25-138)	121.5 (87-167.25)		
Min-Max	28-492	6.2-792		

**Figure 2:**
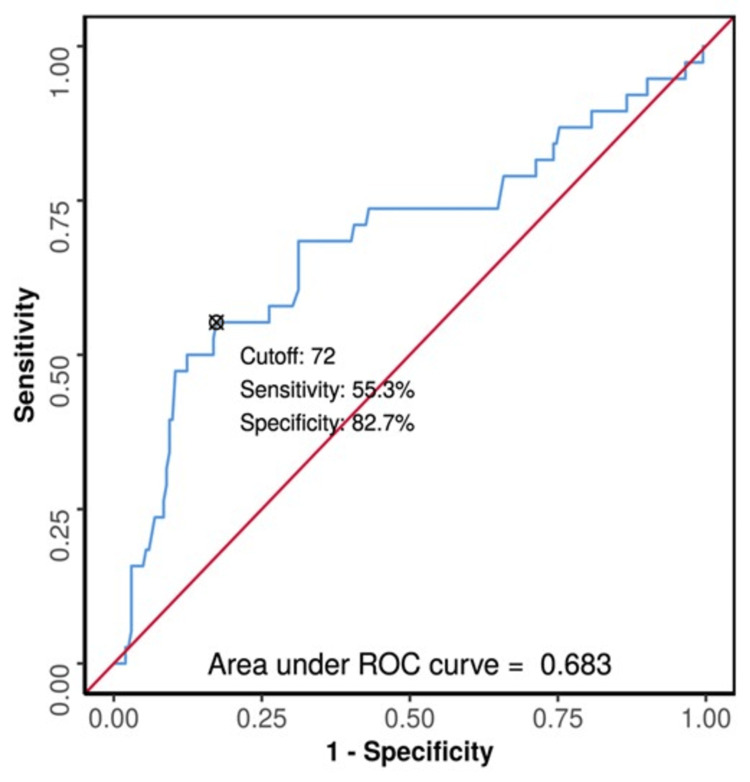
ROC curve analysis showing diagnostic performance of omentin levels in predicting GDM: yes vs. GDM: no (n = 480) The blue line indicates the receiver operating characteristic (ROC) curve representing the diagnostic performance of serum omentin levels; the red diagonal line indicates the line of no discrimination (reference line; AUC=0.5); The black marker (×) indicates the optimal cutoff point (cutoff=72) with corresponding sensitivity and specificity. GDM: gestational diabetes mellitus; AUC: area under the curve

ROC analysis demonstrated that first-trimester omentin levels had moderate ability to discriminate between women who would and would not develop GDM, with an area under the curve (AUROC) of 0.683 (95% CI: 0.577-0.789; p<0.001). A cutoff value of ≤72 ng/mL provided a sensitivity of 55% and specificity of 83%, suggesting better performance in correctly identifying women who remained normoglycemic. The negative predictive value was high at 90.8%, indicating that higher omentin levels were associated with a low likelihood of developing GDM. The overall diagnostic accuracy was 78.3%, and the diagnostic odds ratio was 5.89 (95% CI: 2.82-12.3), supporting the potential role of reduced omentin levels as an early biomarker for GDM (Table 3 and Figure 2).

**Table 3:** ROC curve analysis showing diagnostic performance of omentin levels in predicting GDM: yes vs. GDM: no (n = 480) ROC: receiver operating characteristic curve; AUROC: area under the receiver operating characteristic curve; GDM: gestational diabetes mellitus

Parameter	Value (95% CI)
Cutoff (p-value)	≤72 (<0.001)
AUROC	0.683 (0.577-0.789)
Sensitivity	55.3% (38-71)
Specificity	82.7% (77-88)
Positive predictive value	37.5% (25-51)
Negative predictive value	90.8% (86-95)
Diagnostic accuracy	78.3% (73-83)
Positive likelihood ratio	3.19 (2.11-4.83)
Negative likelihood ratio	0.54 (0.38-0.77)
Diagnostic odds ratio	5.89 (2.82-12.3)

Other maternal variables, including BMI, gravidity, and gestational age at the time of OGTT, were not significantly associated with the development of GDM on either univariate or multivariate analysis. The regression model demonstrated good overall performance, with moderate discriminative ability (C-statistic=0.683) and good calibration (Hosmer-Lemeshow p=0.740). Additionally, the model accounted for a substantial proportion of variability in GDM status (Pseudo-R^2^=0.52). These findings highlight that first-trimester serum omentin level serves as an early predictor of GDM, even after adjusting for other clinical variables (Table 4).

**Table 4:** Multivariable logistic regression analysis for predictors of GDM Model fit: Model χ^2^=18.42, p<0.001; Pseudo-R^2^=0.18; Hosmer-Lemeshow p=0.74; C-statistic (AUROC)=0.683; number included in model=480. GDM: gestational diabetes mellitus; AUROC: area under the receiver operating characteristic curve

Variable	Univariable OR (95% CI)	p-value	Adjusted OR (95% CI)	p-value
BMI (kg/m^2^)	1.09 (0.97-1.23)	0.141	1.04 (0.85-1.26)	0.711
Multigravida vs. primigravida	1.29 (0.63-2.73)	0.496	1.23 (0.41-3.93)	0.711
Serum omentin levels (ng/mL)	0.97 (0.95-0.99)	0.003	0.95 (0.92-0.98)	0.001

The median period of gestation (POG) at delivery was 38 weeks in both groups, with an overall range of 34 to 41 weeks. Further categorization showed that the majority of deliveries occurred between 37 and 39 weeks irrespective of GDM status. Although a slightly higher proportion of early-term deliveries (34-36^+6^ weeks) was observed among women with GDM, the overall gestational age at delivery remained comparable between the two groups.

Hypertensive disorders of pregnancy were among the leading indications for induction of labor in both groups. In women with GDM, gestational hypertension (GHTN) was the most common specific maternal indication (n=19) (25.0%), followed by intrahepatic cholestasis of pregnancy (IHCP) (n=13) (16.7%) and postdatism (n=10) (12.5%). A similar trend was noted in the non-GDM group, where GHTN (n=122) (30.2%), IHCP (n=74) (18.3%), and postdatism (n=67) (16.7%) were the predominant indications. Notably, only two out of 76 (2.63%) women with GDM required induction specifically due to uncontrolled glycaemic status.

Regarding mode of delivery, 34.2% (n=26) of women in the GDM group underwent lower segment caesarean section (LSCS), and 18.4% (n=14) had instrumental deliveries. These rates were slightly higher compared to the non-GDM group, where 32.7% (n=132) underwent LSCS, and 14.4% (n=58) had instrumental deliveries. However, this difference was not statistically significant (χ^2^=0.568, p=0.753). The indications for LSCS were largely similar across both groups, with fetal distress being the most frequent, followed by breech presentation and meconium-stained liquor, each accounting for 15.4% (n=12) in the GDM group.

Neonatal outcomes showed some differences between the groups. The mean birthweight was higher among neonates born to mothers with GDM (2.75 kg) compared to those without GDM (2.64 kg), and this difference reached statistical significance (W=4631.0, p=0.043). Additionally, the distribution of birthweight categories differed significantly (χ^2^=10.388, p=0.006), with a greater proportion of infants weighing more than 3 kg in the GDM group (34.2%, n=26) vs. 16.3% (n=66) in the non-GDM group.

There was no significant association between maternal GDM status and neonatal APGAR scores at either one or five minutes. The median one-minute APGAR score was 8 in both groups, with no meaningful difference observed (W=3815.0, p=0.940).

Birth injuries were relatively uncommon in both groups. Among neonates born to mothers with GDM, four (5.2%) cases were reported, including two (2.63%) cases of caput succedaneum and two (2.63%) of facial bruising. In the non-GDM group, 12 (2.97%) cases were documented, comprising caput succedaneum (n=4) (0.99%), facial bruising (n=2) (0.49%), forceps-related marks (n=2) (0.49%), and scalp lacerations (n=2) (0.49%). Although the proportion was numerically higher in the GDM group (5.2% vs. 2.97%), the difference was not statistically significant (χ^2^=1.414, p=0.242).

Similarly, neonatal intensive care unit (NICU) admissions were slightly more frequent among infants born to mothers with GDM (21.1%) (n=15) compared to those without GDM (18.8%) (n=76). However, this difference did not reach statistical significance (χ^2^=0.104, p=0.747). No consistent statistically significant association was observed between first-trimester serum omentin levels and most fetomaternal outcomes in the study, except for mode of delivery (Table 5).

**Table 5:** Association of omentin levels with fetomaternal outcomes LSCS: low segment cesarean segment; NICU: neonatal intensive care unit; IQR: interquartile range

Fetomaternal outcome	Category	n	Serum omentin level, median (IQR)	Statistical test	p-value
Hypertensive disorder of pregnancy	Yes	106	111.75 (72.75-168)	Mann-Whitney U test	0.454
	No	374	120 (78-160)		
Period of gestation at delivery	<37 weeks	68	120 (46.5-188)	Mann-Whitney U test	0.703
	≥37 weeks	412	118 (82.25-159.5)		
Onset of labor	Induced	158	118 (81.5-160.5)	Mann-Whitney U test	0.932
	Spontaneous	322	120 (66-173)		
Mode of delivery	Vaginal	248	118 (75-158)	Kruskal-Wallis test	0.002
	LSCS	160	130 (97.5-193.5)		
	Instrumental	72	83.5 (55-127.25)		
Indication of LSCS	Fetal distress	48	127 (111.5-152)	Kruskal-Wallis test	0.265
	Breech	12	134 (81-163)		
	Meconium-stained liquor	36	140 (104.25-223)		
	failed induction	22	188(121-231)		
	Scar tenderness	34	145(83-225)		
	Other obstetric indication	8	45(45-45)		
Birth weight	<2 kg	16	139.5 (54-190.25)	Kruskal-Wallis test	0.598
	2-3 kg	372	117.5 (78.5-155)		
	>3 kg	92	127.5 (71.75-176.25)		
NICU admission	Yes	92	122 (77.75-161.75)	Mann-Whitney U test	0.474
	No	388	118 (75.75-161.5)		
Birth injury	Yes	12	89 (53.75-131)	Mann-Whitney U test	0.404
	No	468	119.5 (78-162)		

## Discussion

GDM continues to be a major concern in obstetrics, given its rising incidence and its association with unfavourable fetomaternal outcomes. The condition develops as a result of progressive insulin resistance during pregnancy, coupled with inadequate β-cell compensation. Identifying reliable biomarkers that can predict GDM early in pregnancy is crucial, as this would allow timely interventions to reduce complications and improve long-term outcomes for both mother and child.

In the present study, women diagnosed with GDM were found to have significantly lower serum omentin-1 levels compared to healthy pregnant women. These findings are compatible with earlier reports showing reduced adiponectin concentrations in women with GDM compared to controls. The study population comprised Indian pregnant women aged 18-35 years attending the antenatal clinic at Vardhman Mahavir Medical College and Safdarjung Hospital, New Delhi. The results support previous evidence that reduced omentin-1 concentrations, secreted primarily by visceral adipose tissue, may contribute to insulin resistance and the development of GDM [7]. Omentin-1 is an adipokine produced by both placental tissue and visceral fat, and it has recently gained attention for its possible role in insulin resistance. It is mainly secreted by visceral adipose tissue and acts as a circulating protein with metabolic effects. Experimental evidence suggests that when recombinant omentin-1 is administered, it enhances insulin-mediated glucose uptake in vitro. This indicates that omentin-1 may improve insulin sensitivity and could have an important role in maintaining glucose balance, particularly in conditions associated with impaired insulin action [3,8].

Findings from other studies reinforce this association. Pan et al. observed that circulating omentin concentrations after fasting and two hours post-glucose load were substantially lower in patients with impaired glucose tolerance and in those with recently diagnosed, untreated diabetes compared to healthy controls [9]. Likewise, El-Mesallamy et al. reported decreased circulating omentin levels in patients with type 2 diabetes mellitus after adjusting for age or BMI [10]. Polkowska et al. revealed that circulating omentin concentrations were lower in children with type 1 diabetes mellitus than in control children [11]. These studies suggest that the link between omentin and glucose metabolism extends beyond GDM to other forms of diabetes.

Meta-analyses have also shown that circulating omentin concentrations are lower in patients with GDM than in controls. Interestingly, omentin levels were negatively associated with visceral adipose tissue, despite being primarily expressed in visceral fat. Visceral obesity has been linked to increased release of free fatty acids and inflammatory cytokines into the portal circulation, leading to oxidative stress and insulin resistance [12]. A decrease in omentin secretion from visceral adipose tissue may therefore contribute to the pathogenesis of GDM. Subgroup analyses have highlighted the influence of age and BMI, with differences observed between Caucasian and Asian populations, and between younger and older women with GDM. Omentin levels have also been reported to vary across trimesters, with higher concentrations in the second trimester compared to the third, possibly reflecting placental production and subsequent clearance later in pregnancy [12-17].

Omentin-1 has been shown to reduce C-reactive protein (CRP) and tumor necrosis factor alpha (TNF-α) levels, in addition to its role in glucose metabolism [10]. Diabetes is made easier to develop when omentin dysfunction disrupts glucose homeostasis and increases insulin resistance. By activating AMP-activated protein kinase (AMPK), which in turn triggers the endothelial nitric oxide synthase (eNOS)/NO pathway, Yamawaki et al. showed that omentin reduced TNF-α-induced cyclooxygenase-2 (COX-2) expression [18]. Additionally, omentin promotes endothelium-dependent vasorelaxation mediated by nitric oxide, indicating a function in vascular control [18].

Despite these promising findings, data on omentin concentrations in GDM remain limited. Some studies have reported no substantial differences between GDM patients and controls, whereas others have demonstrated significantly lower maternal circulating omentin concentrations in GDM patients than in healthy pregnant women [19]. They also found an association between maternal obesity in pregnancy and reduced circulating omentin levels. Importantly, no prospective studies evaluating omentin as a predictor of GDM have been published to date.

The current study adds to the growing body of evidence suggesting that lower omentin-1 levels may be associated with GDM, and if it is to be used as an adjunctive or early screening marker rather than a standalone diagnostic tool, it can help in early risk stratification and better management. This study is strengthened by its prospective cohort design, enabling a clear temporal association between first-trimester omentin levels and subsequent GDM. Early biomarker assessment, a well-defined study population with minimal confounding, and a relatively large sample with follow-up until delivery enhance the reliability of findings. Standardized ELISA-based measurements and dual-timepoint OGTT improve diagnostic accuracy, while robust statistical analysis supports the validity of results. Conducted in a tertiary care setting, the findings have strong real-world applicability. However, limitations must be acknowledged. This was a single-center study, and lifestyle factors such as diet and exercise were not considered. Additionally, challenges in obtaining blood samples and information from pregnant women may have influenced the findings. The exploratory fetomaternal outcome analyses were limited by small subgroup sizes and absence of adjusted analyses. Therefore, larger, multicenter, prospective studies are required to confirm whether serum omentin-1 levels in early pregnancy can serve as a reliable biomarker for predicting and prognosing GDM.

## Conclusions

This study demonstrates the potential of omentin-1 as an early biomarker for GDM. Lower omentin-1 levels in women with GDM suggest a possible role in disease development. While the mechanisms are not fully understood, the association with insulin sensitivity points to involvement in maternal metabolic adaptation during pregnancy. These results support the hypothesis that reduced omentin-1 may contribute to insulin resistance and increased GDM risk. Measuring omentin-1 levels could help identify at-risk women early, allowing for timely risk stratification and targeted interventions to improve maternal and perinatal outcomes. First-trimester serum omentin-1 showed a moderate independent association with later GDM development, but further validation is needed before clinical implementation. Ongoing research on additional biomarkers suggests that combining them with omentin-1 may enhance screening, particularly for high-risk Indian women, and enable earlier lifestyle and surveillance interventions before routine OGTT.

## Appendices

Patient proforma

1.      Name:

2.      Case Number :

3.      MRD Number:

4.      Social status (as per modified Kuppuswamy scale): (a) Upper (b) Upper Middle (c) Lower Middle (d) Upper lower (e) Lower

5.      Age:

6.      Address (rural/urban):

7.      Contact number:

8.      Education level: (a) Illiterate (b) Primary School (1st-4th std) (c)Secondary School (till 10th) (d) Intermediate (e)College

9.      Employment: (a) Homemaker (b)Employed

10.  LMP :                                                                               EDD:

11.  Obstetric history: 

a)      GPAL

b)     History of macrosomic baby.                                     Yes/No

c)      History of stillbirth                                                     Yes/No

d)     History of baby with congenital anomaly                  Yes/No

e)      History of instrumental delivery                                Yes/No 

12.  Past history of

·         Diabetes mellitus                                                 Yes/No

·         Hypertension                                                       Yes/No

·         PCOS                                                                   Yes/No

·         Any chronic illness                                              Yes/No

·         Surgical intervention                                           Yes/No

13. Family history of

Diabetes mellitus                                                       Yes/No 

Hypertension                                                             Yes/No 

Any other chronic Illness                                          Yes/No 

Surgical intervention                                                 Yes/No

14. Examination: 

Height.(cm)                         

Weight (kg) 

BMI(kg/m^2^)                         Yes/No

Pallor                                    Yes/No

Oedema.                                Yes/No

Icterus. 

Pulse rate (bpm) 

Blood pressure 

15. USG available: 

16. Investigations: 

CBC 

LFT 

KFT 

SE 

COAG 

TSH 

Urine RM 

Urine CS

17. OGTT done                                Date                     POG                      OGTT Value

At booking visit

First followup at 24- 28 weeks

Second followup at 32-34 weeks

18. Medication History:

19. Fetomaternal outcomes:

Maternal

Hypertensive diseases of pregnancy. If yes; Treatment taken

Labour:                                             Spontaneous/Induced; If induced -indication

POG in labour:                                 

Mode of Delivery:                                    Vaginal/Instrumental delivery/LSCS

Duration of labour:

Fetal

Baby weight(kg):

APGAR score:

Birth injury:                                     Yes/No

NICU admission:        Yes/No
